# A Straightforward Substitution Strategy to Tune BODIPY Dyes Spanning the Near-Infrared Region via Suzuki–Miyaura Cross-Coupling

**DOI:** 10.3390/ma11081297

**Published:** 2018-07-27

**Authors:** Guanglei Li, Yu Otsuka, Takuya Matsumiya, Toshiyuki Suzuki, Jianye Li, Masashi Takahashi, Koji Yamada

**Affiliations:** 1 Division of Materials Science, Graduate School of Environmental Science, Hokkaido University, Sapporo 0860-0810, Japan; li_gl@eis.hokudai.ac.jp (G.L.); y_outsuka214@eis.hokudai.ac.jp (Y.O.); hokkaidou4005@hotmail.co.jp (T.M.); 2 Laboratory of Animal Genetics and Reproduction, Graduate School of Agriculture, Hokkaido University, Sapporo 060-8589, Japan; d-7-shk@eis.hokudai.ac.jp (T.S.); lijianye218@163.com (J.L.); mmasashi@anim.agr.hokudai.ac.jp (M.T.); 3 Global station for Food, Land and Water Resources, Global Institution for Collaborative Research and Education, Hokkaido University, Sapporo 060-0815, Japan; 4 Division of Materials Science, Faculty of Environmental Earth Science, Hokkaido University, Sapporo 0860-0810, Japan

**Keywords:** BODIPY, near-infrared, fluorescence, cellular imaging, Suzuki–Miyaura cross-coupling

## Abstract

In this study, a series of new red and near-infrared (NIR) dyes derived from 4,4-difluoro-4-bora-3a,4a-diaza-s-indacene (BODIPY) were developed by introducing thiophene and its derivatives to the 3- and 5- positions of the dichloroBODIPY core. For the first time, cyclictriol boronates and N-methyliminodiacetic acid (MIDA) boronate were used as organoboron species to couple with 3,5-dichloroBODIPY via the one-step Suzuki–Miyaura cross-coupling. Six kinds of thieno-expended BODIPY dyes were synthesized in acceptable yields ranging from 31% to 79%. All six dyes showed different absorption and emission wavelengths spanning a wide range (c.a. 600–850 nm) in the red and NIR regions with relatively high quantum yields (19–85%). Cellular imaging of 8-(2,6-dimethylphenyl)-re3,5-di(2-thienyl)-BODIPY (dye **1**) was conducted using bovine cumulus cells, and the fluorescence microscopy images indicated that the chromophore efficiently accumulated and was exclusively localized in the cytoplasm, suggesting it could be utilized as a subcellular probe. All six dyes were characterized using 1H-NMR and mass spectrometry.

## 1. Introduction

Long-wavelength dyes, which absorb and emit light in the far-red and near-infrared (NIR) region, have found extensive applications in biology [1] as the spectra in the NIR region has many advantages, such as enhanced sensitivity owing to high contrast and low background noise, deep penetration in tissue, and less damage to organisms. The demand for NIR dyes has greatly stimulated the interest in the design of various novel NIR chromophores that feature optimized properties.

Cyanine is one of the most studied fluorescent dyes. However, its fluorescence tends to be weak and presents low quantum yields except for a few examples [2,3], hence impeding its application in biology. Many NIR dyes have similar drawbacks, while some of them are difficult to synthesize. Therefore, development of alternative NIR dyes and a straightforward modification strategy are imperative.

4,4-difluoro-4-bora-3a,4a-diaza-*s*-indacene (BODIPY) derivatives—also known as boron dipyrromethene—have been widely used over the past two decades due to their outstanding characteristics, such as high molar absorption coefficients, intense fluorescence quantum yields, high stability, and tunable spectroscopic and photophysical properties [4,5]. However, the relatively short wavelength of excitation and emission maxima (generally within 500–600 nm) limit the application of BODIPY chromophores.

Various strategies have been employed to promote the absorption and emission wavelengths of BODIPY dyes to the far-red and NIR regions. Some examples include the extension of π-conjugation length [6,7], rigidification of rotatable moieties [8,9], the introduction of a nitrogen atom in the meso-position to form aza-BODIPY dyes [10,11], and the formation of a “push-pull” motif [12,13].

It has been known that introduction of thiophene subunits to BODIPY results in a remarkable bathochromic shift, and many of them exhibit important optical properties [14,15,16]. Strikingly, in previous research, we found that solvatochromic dyes containing thiophene emitted longer wavelength than other well-known solvatochromic dyes [17], such as *N*-(2-aminoethyl)-4-[5-[4-(dimethylamino)phenyl]-2-oxazolyl]benzenesulfonamide (Dapoxyl SEDA) [18], *N,N*-dimethyl-6-propionyl-2-naphthylamine (PRODAN) [19], and 1-anilinonaphthalene-8-sufonate (ANS) [20]. Therefore, we expect to tune the absorption and emission wavelengths of BODIPYs by introducing different moieties of solvatochromic dyes on the BODIPY core.

The development of NIR dyes generally requires a sophisticated design that involves tedious modification steps. Therefore, it is crucial to develop a simple and systematic approach to tune the wavelengths of BODIPY dyes spanning the NIR region, essentially with high quantum yield.

A few examples of thienyl groups-modified 3,5-disubstituted BODIPY dyes using Stille coupling have been reported [21,22,23,24]. However, organotin compounds are highly toxic, and it is very hard to purify organotin substrates due to their instability on silica or alumina columns. This hinders extensive application of the organotin compounds.

Suzuki–Miyaura cross-coupling—one of the most efficient methods for the construction of C–C bonds—has been employed to couple BODIPY core with phenyl or thienyl subunits [25,26]. To the best of our knowledge, only two examples of modified thiophene units have been reported to couple with 3,5-dihaloBODIPY using Suzuki–Miyaura cross-coupling; however, they were in low yield (~14%) [27]. Basically, there are no available information showing successful introduction of electron donating group-modified thiophene to the 3- and 5- positions of BODIPY core via the cross-coupling reaction. This is mostly due to two reasons: (1) the Suzuki–Miyaura cross-coupling of five-membered boronic acid could be problematic [28,29]; and (2) the first cross-coupling reaction would significantly reduce the reactivity of the remaining halogen.

**Figure 1:**
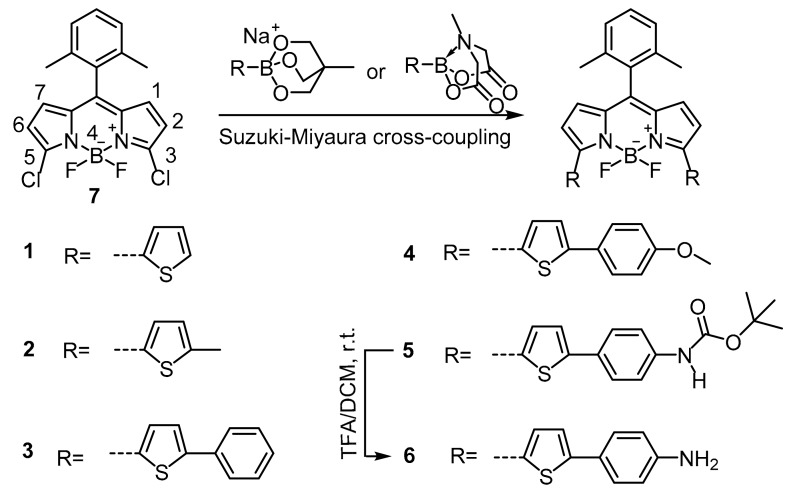
Synthetic scheme to 4,4-difluoro-4-bora-3a,4a-diaza-s-indacene (BODIPY) dyes **1**–**6**. TFA = trifluoroacetic acid.

Herein, we report a straightforward method to introduce thienyl and thienyl derivatives onto a specific 3,5-dichloroBODIPY scaffold via a one-step Suzuki–Miyaura cross-coupling to tune the wavelengths toward the red and NIR regions. Using cyclictriol boronates and N-methyliminodiacetic acid (MIDA) boronate as organoboron species, a set of red and NIR BODIPY dyes were synthesized (Figure 1) in acceptable yields ranging from 31% to 51% under mild reaction conditions, all of which exhibited relatively high quantum yields in the range of 0.19 to 0.85. This is the first time that electron donating group-modified thiophene parts were efficiently introduced onto the 3- and 5- positions of the BODIPY core via Suzuki–Miyaura cross-coupling.

## 2. Materials and Methods

### 2.1. General Experimental

All commercially available solvents and reagents were purchased from suppliers (Sigma-Aldrich Chemical Company (St. Louis, MO, USA), Wako Pure Chemical Industries (Osaka, Japan) or Tokyo Chemical Industry (Tokyo, Japan)), and were used as received unless otherwise noted. Reactions were monitored with high-performance thin-layer chromatography (HPLC; silica gel 60, 0.25mm, F-254, Merck KGaA, Darmstadt, Germany), which were visualized with UV light or/and by a color reaction staining with phosphomolybdic acid solution (5% *w*/*v* in ethanol). Column chromatography was performed using silica gel 60 mesh 230–400 (Wako Pure Chemical Industries, Osaka, Japan).

^1^H-NMR spectra were recorded on a JEOL 400 (400 MHz) spectrometer (JEOL Ltd., Tokyo, Japan) at room temperature. Chemical shifts were expressed in parts per million (ppm) relative to the standard reference tetramethylsilane (TMS) (0 ppm). Coupling constants (*J*) were expressed in Hertz. Mass spectra were carried out on a Thermo Scientific Exactive (Thermo Fisher Scientific K.K., Tokyo, Japan) under electrospray ionization (ESI) or atmospheric pressure chemical ionization (APCI) conditions. UV-visible absorption spectra were performed on a JASCO V-560 spectrophptometer (JASCO Corporation, Tokyo, Japan), and fluorescence spectra and fluorescence quantum yields were measured with Hamamatsu Photonics Quantaurus-QY Absolute PL quantum yield spectrometer C11347 (Hamamatsu Photonics K.K., Hamamatsu, Japan); fluorescence microscopy images were carried out on Leica DMi 8 fluorescence microscope (Leica Camera AG, Wetzlar, Germany).

### 2.2. Synthesis of BODIPY Dyes ***1**–**6***

#### 2.2.1. General Procedure: Synthesis of Dyes **1**–**4**

The starting material—3,5-dichloroBODIPY core (compound **7**)—was synthesized according to the previously published method [30]. Compound **7** (1 equiv), Palladium (II) acetate [Pd(OAc)_2_] (10 mol %), 2-Dicyclohexylphosphino-2′,6′-dimethoxybiphenyl (SPhos) (20 mol %), CuCl (0.4 equiv) and respective boronates (3 equiv) were placed in a two-necked round bottom flask. Prior to the addition of *N,N*-dimethylformamide (DMF), the flask was purged with N_2_ three times. The reaction was stirred at 60 °C for 24 h, and the mixture was allowed to cool to room temperature, then extracted with ethyl acetate and washed with H_2_O and brine (saturated NaCl solution) successively. The organic layer was collected and dried over Na_2_SO_4_ and evaporated to dryness under reduced pressure. The crude sample was purified by silica gel column chromatography using eluent gradients with the eluent pair hexane/ethyl acetate.

##### Synthesis of 8-(2,6-Dimethylphenyl)-3,5-di(2-thienyl)-BODIPY (**1**)

Prepared according to the general procedure using compound **7** (91 mg, 0.25 mmol), (2-thiophene) cyclic-triolborate sodium salt (175 mg, 0.75 mmol), Pd(OAc)_2_ (6 mg, 0.025 mmol), SPhos (21 mg, 0.05 mmol), CuCl (10 mg, 0.1 mmol) and DMF (2.5 mL) to afford the desired product as a dark green solid (54 mg, 47%). ^1^H-NMR (400 MHz, CDCl_3_): δ = 8.24 (dd, *J* = 3.8 Hz, *J* = 1 Hz, 2H), δ = 7.49 (dd, *J* = 5.4 Hz, *J* = 1 Hz, 2H), δ = 7.31~7.27 (m, 1H), δ = 7.21 (dd, *J* = 4.9 Hz, *J* = 3.9 Hz, 2H), δ = 7.15 (d, *J* = 7.8 Hz, 2H), δ = 6.76 (d, *J* = 4.3 Hz, 2H), δ = 5.54 (d, *J* = 3.9 Hz, 2H), δ = 2.20 (s, 6H). ESI-FTMS (*m*/*z*) Calculated for C_25_H_19_BF_2_N_2_S_2_: 460.11; found [M + H]^+^: 461.12.

##### Synthesis of 8-(2,6-Dimethylphenyl)-3,5-di(5-methyl-2-thienyl)-BODIPY (**2**)

Prepared according to the general procedure using compound **7** (182 mg, 0.5 mmol), 2-(5-methylthiophene) cyclic-triolborate sodium salt (372 mg, 1.5 mmol), Pd(OAc)_2_ (11 mg, 0.05 mmol), SPhos (41 mg, 0.1 mmol), CuCl (20 mg, 0.2 mmol) and DMF (5 mL) to afford the desired product as a dark green solid (125 mg, 51%). ^1^H-NMR (400 MHz, CDCl_3_): δ = 8.01 (d, *J* = 3.4 Hz, 2H), δ = 7.33–7.24 (m, 1H), δ = 7.13 (d, *J* = 7.3 Hz, 2H), δ = 6.86 (dd, J = 3.9 Hz, *J* = 1.0 Hz, 1H), δ = 6.67 (d, *J* = 4.4 Hz, 2H), δ = 6.48 (d, *J* = 4.4 Hz, 2H), δ = 2.56 (s, 6H), δ = 2.19 (s, 6H). ESI-FTMS (*m*/*z*) Calculated for C_27_H_23_BF_2_N_2_S_2_: 488.14; found [M + H]^+^: 489.15.

##### Synthesis of 8-(2,6-Dimethylphenyl)-3,5-di[(5-phenyl(2-thienyl)]-BODIPY (**3**)

Prepared according to the general procedure using compound **7** (91 mg, 0.25 mmol), **S2** (see supplementary materials) (240 mg, 0.75 mmol), Pd(OAc)_2_ (6 mg, 0.025 mmol), SPhos (21 mg, 0.05 mmol), CuCl (10 mg, 0.1 mmol) and DMF (2.5 mL) to afford the desired product as a dark green solid (54 mg, 35%). ^1^H-NMR (400 MHz, CDCl_3_): δ = 8.27 (d, *J* = 3.9 Hz, 2H), δ = 7.69 (d, *J* = 7.4 Hz, 4H), δ = 7.45-7.40 (m, 6H), δ = 7.34 (d, *J* = 7.3 Hz, 2H), δ = 7.31~7.26 (m, 1H), δ = 7.16 (d, *J* = 7.6 Hz, 2H), δ = 6.80 (d, *J* = 4.3 Hz, 2H), δ = 6.54 (d, *J* = 4.3 Hz, 2H), δ = 2.22 (s, 6H). ESI-FTMS (*m*/*z*) Calculated for C_37_H_27_BF_2_N_2_S_2_: 612.17; found [M]^+^: 612.17.

##### Synthesis of 8-(2,6-Dimethylphenyl)-3,5-di[(5-phenyl(2-thienyl)]-BODIPY (**4**)

Prepared according to the general procedure using compound **7** (182 mg, 0.5 mmol), **S6** (see supplementary materials) (510 mg, 1.5 mmol), Pd(OAc)_2_ (11 mg, 0.05 mmol), SPhos (41 mg, 0.1 mmol), CuCl (20 mg, 0.2 mmol) and DMF (5 mL) to afford the desired product as a purple solid (129 mg, 38%). ^1^H-NMR (400 MHz, CDCl_3_): δ = 8.24 (d, *J* = 4.1 Hz, 2H), δ = 7.62 (d, *J* = 8.8 Hz, 4H), δ = 7.33 (d, *J* = 4.0 Hz, 2H), δ = 7.30~7.26 (m, 1H), δ = 7.15 (d, *J* = 7.7 Hz, 2H), δ = 6.95 (m, 1H), δ = 6.78 (d, *J* = 4.4 Hz, 2H), δ = 6.52 (d, *J* = 4.4 Hz, 2H), δ = 3.86 (s, 6H), δ = 2.21 (s, 6H). ESI-FTMS (*m*/*z*) Calculated for C_39_H_31_BF_2_N_2_O_2_S_2_: 672.19, found [M]^+^: 672.19.

#### 2.2.2. Synthesis of 8-(2,6-Dimethylphenyl)-3,5-di[5-(4-BOC-aminophenyl)-2-thienyl)]-BODIPY (**5**)

Compound **7** (25 mg, 0.07 mmol), Pd(OAc)_2_ (2 mg, 0.007 mmol), 2-Dicyclohexylphosphino-2′,4′,6′-triisopropylbiphenyl (XPhos) (7 mg, 0.014 mmol), and MIDA boronate **S10** (see supplementary materials) (88 mg, 0.21 mmol) were placed in a two-necked round bottom flask. The flask was then purged with N_2_ three times before addition of dioxane (5 mL). The mixture was stirred at ambient temperature for 5 min. Then, K_3_PO_4_ (0.5 M, 1 mL) was added, and the reaction mixture was stirred at 60 °C for two days. The mixture was allowed to cool to room temperature, extracted with ethyl acetate, washed with H_2_O and brine successively. The organic layer was collected, dried over Na_2_SO_4_ and evaporated to dryness under reduced pressure. The crude sample was purified by silica gel column chromatography (ethyl acetate: hexane 1: 4) to afford dark green solid **5** (18 mg) in 31% yield. ^1^H-NMR (400 MHz, CDCl_3_): δ = 8.23 (d, *J* = 4.1 Hz, 2H), δ = 7.61 (d, *J* = 8.6 Hz, 4H), δ = 7.42 (d, *J* = 8.4 Hz, 4H), δ = 7.36 (d, *J* = 4.0 Hz, 2H), δ = 7.30~7.24 (m, 1H), δ = 7.15 (d, *J* = 7.8 Hz, 2H), δ = 6.78 (d, *J* = 4.4 Hz, 2H), δ = 6.55 (bs, 2H), δ = 6.52 (d, *J* = 4.4 Hz, 2H), δ = 2.21 (s, 6H), δ = 1.54 (s, 18H). ESI-FTMS (*m*/*z*) Calculated for C_47_H_45_BF_2_N4O_4_S_2_: 842.29, found [M − H]^+^: 841.29.

#### 2.2.3. Synthesis of 8-(2,6-Dimethylphenyl)-3,5-di[5-(4-aminophenyl)-2-thienyl)]-BODIPY (**6**)

Dye **5** (26 mg, 0.03mmol) dissolved in 1 mL dichloromethane (DCM) was stirred under N_2_ for 5 min at 0 °C, followed by dropwise addition of TFA (60 μL), and the reaction mixture was stirred for 24 h at ambient temperature. After cooling to 0 °C, the reaction was quenched with 1 mL saturated solution of NaHCO_3_, extracted with ethyl acetate, washed with H_2_O and brine successively. The organic layer was collected, dried over Na_2_SO_4_, and evaporated to dryness under reduced pressure. The crude sample was purified by silica gel column chromatography (CHCl_3_ 100%) to afford black solid **6** (11 mg) in 79% yield. ^1^H-NMR (400 MHz, CDCl_3_): δ = 8.23 (d, *J* = 4.2 Hz, 2H), δ = 7.49 (d, *J* = 8.4 Hz, 4H), δ = 7.30~7.24 (m, 3H), δ = 7.14 (d, *J* = 7.7 Hz, 2H), δ = 6.76 (d, *J* = 4.3 Hz, 2H), δ = 6.70 (d, *J* = 8.4 Hz, 4H), δ = 6.49 (d, *J* = 4.3 Hz, 2H), δ = 2.20 (s, 6H). ESI-FTMS (*m*/*z*) Calculated for C_37_H_29_BF_2_N_4_S_2_: 642.19; Found [M − H]^+^: 641.19.

### 2.3. Collection and Culture of Bovine Cumulus Cells

Bovine ovaries were obtained from a local slaughterhouse. The ovaries were washed in a sterile solution of saline containing 10 IU/mL of penicillin and streptomycin. After oocytes pick up, cumulus cells remaining in the follicular fluid were used for the experiment. Collected cumulus cells were transferred to 8 well chamber slide (Watson, Tokyo, Japan) filled with Dulbecco’s minimal essential medium (DMEM) containing 5% fetal bovine serum (FBS). Then, cells were cultured for 1–2 days at 38.0 °C in a humidified atmosphere of 95% air and 5% CO_2_. After reaching 70–80% of confluency, each well was washed and replaced with fresh DMEM containing 5% FBS.

### 2.4. Cellular Staining Study of Dye ***1***

Cell staining was carried out using dye **1**. Stock dye solution (10 μM in dimethyl sulfoxide) was diluted with DMEM containing 5% FBS to make final concentration at 0.2 μM. Hoechst 33242 (Thermo Fisher Scientific, Waltham, MA, USA) was also added to the same medium to stain the nuclei. After 1 h incubation at 38.0 °C in 5% CO_2_ incubator, the culture medium was removed and the cells were washed with PBS, and fresh culture medium was added to each well. Images were acquired using a fluorescent microscope with DAPI and TexasRed (TX2) filter cubes.

## 3. Results and Discussion

### 3.1. Synthesis of Dyes ***1**–**6***

Introduction of a 2,6-dimethylphenyl moiety at the meso-position of BODIPY core is an efficient way to increase the fluorescence quantum yield by restricting the internal rotation of the phenyl ring caused by the two ortho methyl groups [31].

Suzuki–Miyaura cross-coupling often gives undesirable result for heteroaromatic boronate due to the accelerated hydrolytic B–C bond cleavage in basic aqueous condition during Suzuki–Miyaura cross-coupling [32,33,34]. Cyclictriol boronate is superior as it can undergo Suzuki–Miyaura cross-coupling even in the absence of a base [35], which would diminish the competitive hydrolytic B–C bond cleavage during the reaction. In addition, cyclictriol boronate is an air- and water-stable boron reagent. This easy-handling boronate, which has extremely high nucleophilicity [36] and good solubility in organic solvents, tends to have high reactivity coupling with halogens [37]. Thus, the cyclic boronate was applied for this study.

It has been demonstrated that electron-rich and bulky ligands could facilitate the Suzuki–Miyaura cross-coupling by increasing the rate of the oxidative addition and reductive elimination process [38,39]. Moreover, the addition of copper(І) can promote Suzuki–Miyaura cross-coupling [35,40]. Based on this information, dyes **1**–**4** were synthesized under the optimized conditions and isolated in relatively high yields ranging from 35% to 51%, considering the fact that electron-neutral or electron-rich aryl chlorides have low reactivity and are regarded as inactivated chlorides [41]. In this disubstitution reaction, an electron donating group at the first substitution step inhibited the reactivity of the monosubstituted intermediates as the donating groups increased the electron density via a resonance donating effect.

The MIDA boronate, obtained from boronic acid [42,43], was also synthesized as an alternative boron reagent for the modification of the BODIPY core. MIDA boronate ester is stable under various conditions and can slowly release the corresponding boronic acid in mild basic aqueous solution [42]. This ester is more effective than the corresponding boronic acid when coupling with chlorides under mild basic aqueous condition [44]. Using the optimized conditions, dye **5** was produced with a yield of 31%, and dye **6** was obtained a 79% yield via reaction of dye **5** with trifluoroacetic acid at room temperature.

Compared to the reported methods using Stille coupling [21,22,23,24], the synthetic method described here is advantageous as the reactions were conducted in mild condition (60 °C) with competitive yields, and no toxic regent is involved in the reactions.

### 3.2. Spectroscopic and Photophysical Properties of Dyes ***1**–**6***

**Figure 2:**
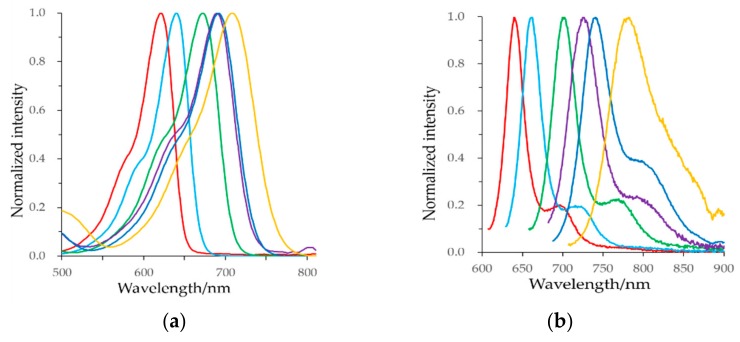
Normalized absorption (**a**) and fluorescence (uncorrected) (**b**) spectra of BODIPYs **1**–**6** in dichloromethane (DCM) (red, **1**; light blue, **2**; green, **3**; purple, **4**; blue, **5**; and yellow, **6**, respectively).

The spectroscopic characterization of these BODIPY dyes was performed in DCM as shown in Figure 2 and are summarized in Table 1. The absorption and emission maxima could be greatly affected by the introduction of electron donating substitution at 3,5-position [4,5,8]. The absorption and emission bands of dyes **1**–**6** were in the red to NIR regions. The absorption spectra showed a strong S_0_ to S_1_ transition with absorption maxima varying between 621 nm and 708 nm, and the shoulder peak located at shorter wavelength was ascribed to the S_0_ to S_2_ transition. The emission bands were the mirror images of the absorption ones with moderate Stokes shift; emission maxima were in the range of 640–780 nm.

Unexpectedly, the methyl at the α-position of the thiophene in dye **2** induced appreciable bathochromic shifts of 4.78 × 10^2^ cm^−1^ in the absorption and 5.20 × 10^2^ cm^−1^ in the emission. A similar phenomenon was also found in a previous report, i.e., one additional methyl group red-shifted the absorption and emission maxima by about 2.01 × 10^2^ cm^−1^ and 1.64 × 10^2^ cm^−1^, respectively [45]. It violated the general assertion that as a weak electron donating group, the methyl group generally has little effect on wavelengths of fluorescent dyes.

The extension of π-conjugation also led to a remarkable bathochromic shift (12.23 × 10^2^ cm^−1^ and 13.60 × 10^2^ cm^−1^ for the absorbance and emission band, respectively), dye **3** showed an absorption maximum at 672 nm and the emission at 701 nm. As expected, further extension of the π-conjugation and the addition of electron donating group increased the bathochromic shift. As a result, the absorption and the emission maxima of dye **6** were shifted to 708 nm and 780 nm, respectively.

Interestingly, dyes **4** and **5** showed similar absorption maximum (c.a. 690 nm), but their emission maxima were at 725 nm and 740 nm, respectively. The difference between the emission maxima may arise from the geometry relaxation of the dyes upon photoexcitation [46]. We anticipate that compared to the methoxy group, the bulky *tert*-butyloxycarbonyl protecting amine group could induce larger geometry relaxation at the excited state (S_1_ state), and the decreased energy gap will produce a larger Stokes shift. The increased wavelengths were likely caused by the combined effects of the extension of the π-electrons delocalization, the strength of the π-electron donors, and the sulfur atoms. Therefore, the wavelengths of the synthesized dyes could be fine-tuned using a stronger donating group and/or achieving a longer extension of the π-conjugation, giving rise to the wavelengths that shifted to lower energy.

Fluorescence quantum yield is one of the most important parameters to evaluate fluorophores. It directly reflects the efficiency of the conversion of absorbed photons into emitted ones. In addition, many far-red and NIR chromophores encounter low fluorescence quantum yield [3,16]. To our delight, the bathochromic shift was achieved without compromising the fluorescence quantum yield: dyes **1**–**5** were quite high in the range of 0.53 to 0.85. Although for dye **6**, which was synthesized by removing the BOC group from dye **5**, a decrease in the fluorescence quantum yield was observed (0.19 in DCM), it still remained at a moderate level. This decrease may result from the formation of hydrogen bonds between the amine groups and hence invoke rapid quenching of the singlet state through intramolecular charge transfer (ICT) [47,48]. On the whole, no obvious fluorescence quenching was observed, indicating that the modification method is a feasible way to develop NIR BODIPY dyes.

### 3.3. Cellular Imaging of Dye ***1***

**Figure 3:**
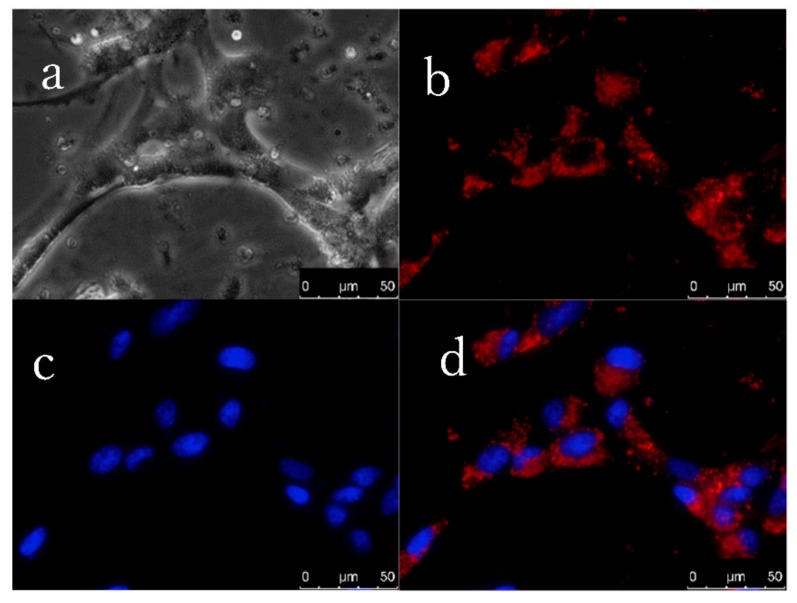
Fluorescence colocalization images of dye **1** and Hoechst 33242 in live bovine cumulus cells. (**a**) bright field; (**b**) cellular uptake of dye **1** in the cytoplasm, TX2 filters (BP 560/40, BP 645/75); (**c**) Hoechst stained nuclei region, DAPI filters (BP 350/50, BP 460/50); (**d**) overlay of b/c.

To assert the performance of our BODIPY dyes in practical use, cellular imaging was conducted. Dye **1** was selected as the representative dye to study cellular uptake for its inherent high fluorescent quantum yield, which would enable facile visualization with fluorescence microscopy in the red region. To simplify the cellular observation, a dual staining experiment was performed. 2 μM aqueous dye **1** and Hoechst were incubated with bovine cumulus cells for 1 h at 38 °C. Excitation and observation of dye 1 and Hoechst were achieved using TX2 and DAPI filter cubes, respectively. Dye **1** and Hoechst showed efficient accumulation in the cells, as shown in Figure 3b,c. Dye **1** appeared to be accumulated in the cytoplasm (red color) of the cell, but not in the nucleus (black area), as shown in Figure 3b. We further merged these two images, and an image of clear contrast (Figure 3d) was obtained. As we know that Hoechst specifically stains the nuclei other than the cytoplasm, the images indicated that dye **1** was exclusively localized in the cytoplasm; this suggests dye **1** has prospect to be used as a subcellular probe.

## 4. Conclusions

In this paper, a facile approach to develop red and NIR BODIPY dyes was proposed. Six different red and NIR BODIPY dyes were rationally designed and synthesized from readily available 3,5-dichloroBODIPY via a one-step Suzuki–Miyaura cross-coupling. Dyes **1**–**6** showed different absorption (621–708 nm) and emission (640–780 nm) with relatively high quantum yields (19–85%) in DCM. Extension of π-conjugation and the addition of electron donating group increased the bathochromic shift. Dye **6** bearing amine groups exhibited the longest absorption maximum λ_abs_ (708 nm) and emission maximum λ_em_ (780 nm) and the longest Stokes shift (72 nm). The fluorescence microscope images showed efficient uptake of dye **1** with bovine cumulus cells, and the dye was exclusively localized in cytoplasm rather than the nucleus.

## Figures and Tables

**Table 1:** Spectroscopic data of dyes **1**–**6** in dichloromethane (DCM).

Dye	λ_abs_/nm	λ_em_/nm	Φ_f_	ε/M^−1^ cm^−1^	Stokes Shift/cm^−1^
**1**	621	640	0.85	69,000	4.78 × 10^2^
**2**	640	662	0.81	66,000	5.19 × 10^2^
**3**	672	701	0.71	75,000	6.16 × 10^2^
**4**	689	725	0.68	78,000	7.21 × 10^2^
**5**	691	740	0.53	82,000	9.58 × 10^2^
**6**	708	780	0.19	72,000	13.0 × 10^2^

## Supplementary Materials

The following are available online at http://www.mdpi.com/1996-1944/11/8/1297/s1, Figure S1: Synthesis scheme of dyes **1**–**4**, Figure S2: Synthesis scheme of dyes **5** and **6**, Figure S3: Synthesis scheme of **S2**, Figure S4: Synthesis scheme of **S6**, Figure S5: Synthesis scheme of **S10**.
