# Supplementary material for: A Straightforward Substitution Strategy to Tune BODIPY Dyes Spanning the Near-Infrared Region via Suzuki–Miyaura Cross-Coupling
Source: Materials (Basel). 2018 Jul 27;11(8):1297. doi: 10.3390/ma11081297 (PMC6117675; doi:10.3390/ma11081297)
Supplement: Supplementary file 1 [file materials-11-01297-s001.pdf]

Supplementary Information

A Straightforward Substitution Strategy to Tune BODIPY Dyes Spanning the Near-Infrared Region via Suzuki–Miyaura Cross-Coupling

Guanglei Li, Yu Otsuka, Takuya Matsumiya, Toshiyuki Suzuki, Jianye Li, Masashi Takahashi and Koji Yamada

1. Synthetic Procedures of dyes 1–6

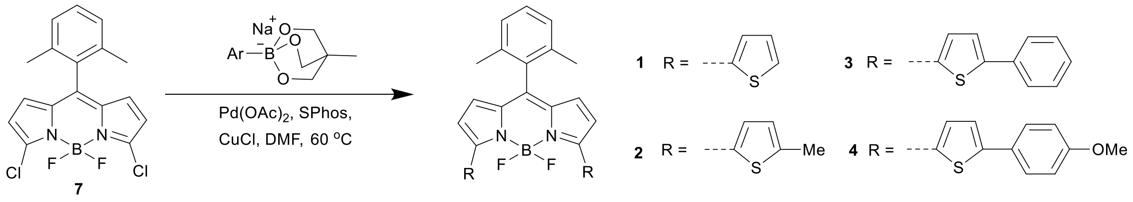

Figure S1. Synthesis scheme of dyes **1–4**

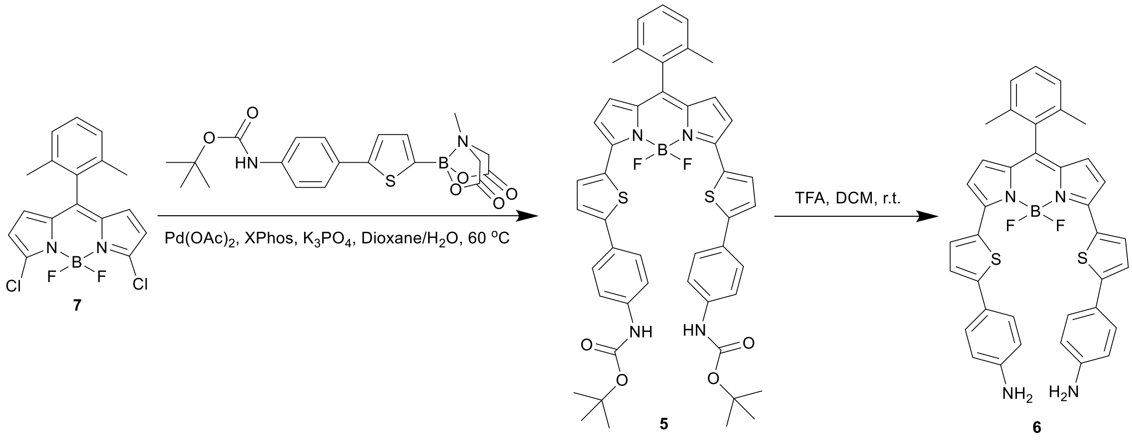

Figure S2. Synthesis scheme of dyes **5** and **6**

2. Synthetic Procedures and characterization of cyclictriol boronates (S2 and S6) and MIDA boronate (S10)

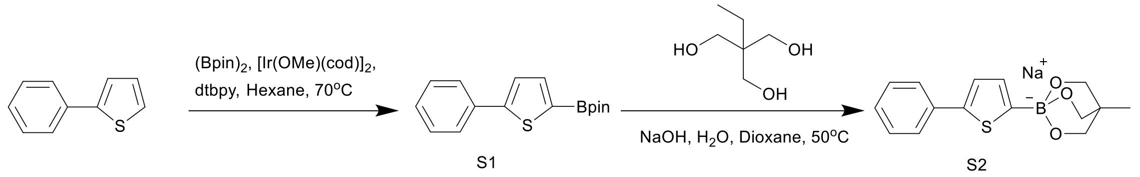

Figure S3. Synthesis scheme of **S2**

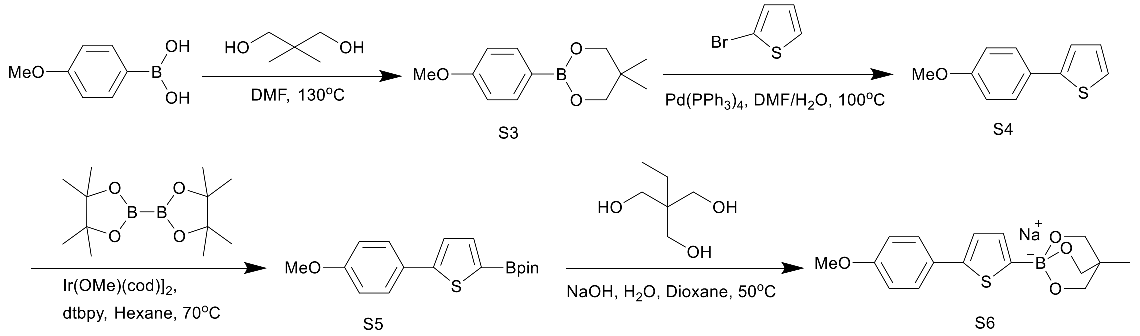

Figure S4. Synthesis scheme of **S6**

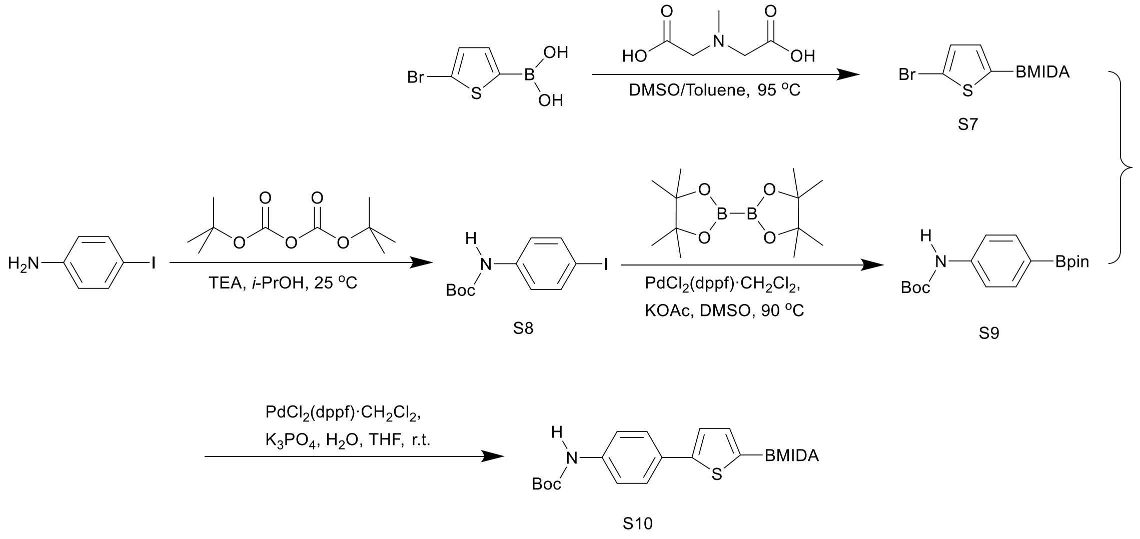

Figure S5. Synthesis scheme of **S10**

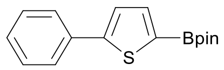

4,4,5,5-Tetramethyl-2-(5-phenylthiophen-2-yl)-1,3,2-dioxaborolane (S1)

2-Phenylthiphene (400 mg, 2.5 mmol, 1 equiv), Bis(pinacolato)diboron (1.268 g, 5.0 mmol, 2 equiv), $[Ir(OMe)(cod)]_2$ (33 mg, 0.05 mmol, 2 mol%) and dtbpy (33.5 mg, 0.12 mmol, 5 mol%) were dissolved in hexane (150 mL), flushed with nitrogen gas three times in an oven-dried two-neck flask. The reaction mixture was stirred at 70 °C for 1 h. After cooling to room temperature, the mixture was extracted with EtOAc, washed with H_2O and brine successively. The organic layer was collected, dried over Na_2SO_4, filtered, and then concentrated. The crude sample was purified by silica gel column chromatography (EtOAc: hexane 1: 40) to give target compound **S1** as grey solid (421 mg, 59%).

^{1}H-NMR (400 MHz, $CDCl_3$): δ = 7.65 (d, *J* = 7.4 Hz, 2H), δ = 7.61 (d, *J* = 3.4 Hz, 1H), δ = 7.40~7.37 (m, 3H), δ = 7.30 (t, *J* = 7.1 Hz, 1H), δ = 1.37 (s, 12H). APCI-FTMS (*m*/*z*) calcd for $C_{16}H_{19}BO_2S$: 286.12; Found $[M+H]^+$: 287.16.

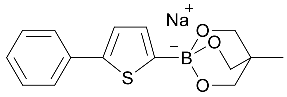

2-(5-phenylthiophene) Cyclic-triolborate Sodium salt (S2)

S1 (100 mg, 0.35 mmol, 1 equiv), 1,1,1-tris(hydroxymethyl)ether (42 mg, 0.35 mmol, 1 equiv), crushed NaOH (14 mg, 0.35 mmol, 1 equiv) were placed in a two-necked round bottom flask. The flask was then purged with nitrogen gas three times before addition of dioxane (5 mL) and H_2O (19 μL, 10.5 mmol, 3 equiv). The mixture was stirred at 50 °C for 24 h. After the reaction was complete, the mixture was allowed to cool to room temperature, and the white precipitate was collected by filtration, washed with diethyl ether (3 × 10 mL) to give target compound **S2** as grey solid (73 mg, 68%).

^{1}H-NMR (400 MHz, DMSO): δ = 7.53 (d, *J* = 7.3 Hz, 2H), δ = 7.32 (t, *J* = 7.7 Hz, 2H), δ = 7.18 (d, *J* = 3.3 Hz, 1H), δ = 7.14 (t, *J* = 7.4 Hz, 1H), δ = 6.69 (d, *J* = 3.2 Hz, 1H), δ = 3.56 (s, 6H), δ = 0.48 (s, 3H). ESI-FTMS (*m*/*z*) calcd for $C_{15}H_{16}BO_3SNa$: 310.08; Found $[M-Na]^+$: 287.09.

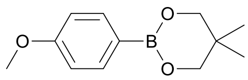

5,5-Dimethyl-2-(4-methoxyphenyl) -1,3,2-dioxaborinane (S3)

p-methoxyphenyl boronic acid (1.00 g, 6.6 mmol, 1 equiv), 2,2-Dimethyl-1,3-propanediol (0.822 g, 7.9 mmol, 1.2 equiv) were dissolved in DMF (20 mL), flushed with nitrogen gas three times in an oven-dried two-neck flask. The mixture was stirred at 130 °C for 2 h. After cooling to room temperature, the mixture was extracted with EtOAc, washed with H_2O and brine successively. The organic layer was collected, dried over Na_2SO_4, filtered, and then

concentrated. The crude sample was purified by silica gel column chromatography (EtOAc: Hexane 1: 40) to give target compound **S3** as white solid (1.50 g, 100%). APCI-FTMS (*m/z*) calcd for $C_{12}H_{17}BO_3$: 220.13; Found $[M+H]^+$: 221.14.

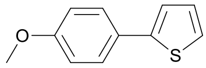

2-(4-methoxyphenyl)-thiophene (S4)

S3 (1.50 g, 6.82 mmol, 1 equiv), bromothiophene (1.334 g, 8.18 mmol, 1.2 equiv), CS_2CO_3 (4.442 g, 18.36 mmol, 2 equiv), $Pd(PPh_3)_4$ (394 mg, 0.34 mmol, 5 mol%) were placed in a two-necked round bottom flask. The flask was then purged with nitrogen gas three times before addition of DMF (20 mL) and H_2O (2 mL). The mixture was stirred at 100 °C for 3 h. After the reaction was complete, the mixture was allowed to cool to room temperature, extracted with EtOAc, washed with H_2O and brine successively. The organic layer was collected, dried over Na_2SO_4, filtered, and then concentrated. The crude sample was purified by silica gel column chromatography (DCM: hexane 1: 5) to give target compound **S4** as yellowish solid (1.185 g, 91%).

^{1}H-NMR (400 MHz, $CDCl_3$): δ = 7.72 (dt, *J* = 8.5 Hz, *J* = 2.3 Hz, 2H), δ = 6.87 (dt, *J* = 8.7 Hz, *J* = 2.4 Hz, 2H), δ = 3.80 (s, 3H), δ = 3.73 (s, 4H), δ = 1.00 (s, 6H). APCI-FTMS (*m/z*) calcd for $C_{11}H_{10}OS$: 190.05; Found $[M+H]^+$: 191.05.

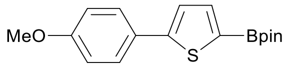

4,4,5,5-Tetramethyl-2-[5-(4-methoxylphenyl)-2-thienyl)-1,3,2-dioxaborolane (S5)

The synthesis of **S5** (light green solid, 660 mg, yield 66%) is the same as the synthesis of **S1**, using **S4** (600 mg, 3.15 mmol, 1 equiv), Bis(pinacolato)diboron (916 mg, 3.78 mmol, 1.2 equiv), $[Ir(OMe)(cod)]_2$ (42 mg, 0.06 mmol, 2 mol%) and dtbpy (42 mg, 0.16 mmol, 5 mol%) and hexane (150 mL).

^{1}H-NMR (400 MHz, $CDCl_3$): δ = 7.59-7.56 (m, 3H), δ = 7.27 (d, *J* = 3.7 Hz, 1H), δ = 6.91 (d, *J* = 8.9 Hz, 2H), δ = 3.83 (s, 3H), δ = 1.36 (s, 12H). ESI-FTMS (*m/z*) calcd for $C_{17}H_{21}BO_3S$: 316.13; Found $[M+H]^+$: 317.14.

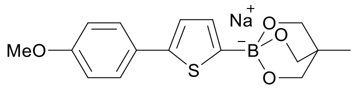

2-[5-(4-methoxylphenyl) thiophene] Cyclic-triolborate Sodium salt (S6)

The synthesis of **S6** (gray solid, 584 mg, yield 82%) is the same as the synthesis of **S2,** using **S5** (660 mg, 2.09 mmol), 1,1,1-tris(hydroxymethyl)ether (251 mg, 2.09 mmol, 1 equiv), crushed NaOH (84 mg, 2.09 mmol, 1 equiv) dioxane (20 mL) and H_2O (113 µL, 6.3 mmol, 3 equiv).

^{1}H-NMR (400 MHz, DMSO): δ = 7.44 (d, *J* = 8.7 Hz, 2H), δ = 7.03 (d, *J* = 2.9 Hz, 1H), δ = 6.90

(d, *J* = 8.8 Hz, 2H), δ = 6.64 (m, *J* = 2.9 Hz, 1H), δ = 0.47 (s, 3H). ESI-FTMS (*m*/*z*) calcd for $C_{16}H_{18}BO_4SNa$: 340.09; Found $[M-H]^+$: 339.20.

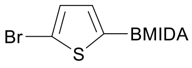

4-bromothiophene-2-boronic acid MIDA ester (S7)

The 4-bromothiophene-2-boronic acid (1.0 g, 4.83 mmol, 1 equiv), *N*-methyliminodiacetic acid (MIDA, 0.78 g, 5.31 mmol, 1.1 equiv) were dissolved in DMSO (3 mL) and Toluene (30 mL), flushed with nitrogen gas three times in an oven-dried two-neck flask. The flask was fitted with a Dean–Stark trap, and the mixture was stirred at 95 °C for 20 h, tolune was removed under reduced pressure. H_2O was added and the resulting precipitate was collected by filtration. The precipitate was washed with H_2O (3 × 30 mL) and diethyl ether (3 × 30 mL) to give the target compound as white crystalline solid (1.45 g, 94%).

^{1}H-NMR (400 MHz, ACETONE-6d): δ = 7.19 (d, *J* = 3.5 Hz, 1H), 7.13 (d, *J* = 3.5 Hz, 1H), δ = 4.39 (d, *J* = 17 Hz, 2H), δ = 4.20 (d, *J* = 17 Hz, 2H), δ = 2.92 (s, 3H). ESI-FTMS (*m*/*z*) calcd for $C_{20}H_{23}BN_2O_6S$: 316.95; Found $[M+H]^+$: 317.94.

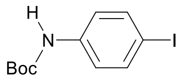

***tert*-butyl (4-iodophenyl) carbamate (*N*-Boc-4-Iodoaniline) (S8)**

The 4-Iodoaniline (2.0 g, 9.13 mmol, 1 equiv), di-*tert*-butyl dicarbonate ($(Boc)_2O$, 2.39 g, 10.96 mmol, 1.2 equiv), Triethylamine (1.08 g, 18.26 mmol, 2 equiv) were placed in a two-necked round bottom flask. The flask was then purged with nitrogen gas three times before addition of *i*-PrOH (50 mL). The mixture was stirred at 25 °C for 20 h. After the reaction was complete, the mixture was extracted with EtOAc, washed with H_2O and brine successively. The organic layer was collected, dried over Na_2SO_4, filtered, and then concentrated. The crude sample was purified by silica gel column chromatography (EtOAc: hexane 1: 9) to yield the target compound as white solid (2.83 g, 99%).

^{1}H-NMR (400 MHz, $CDCl_3$): δ = 7.58 (d, *J* = 8.9 Hz, 2H), δ = 7.14 (d, *J* = 8.7 Hz, 2H), δ = 6.44 (bs, 1H), δ = 1.51 (s, 9H). ESI-FTMS (*m*/*z*) calcd for $C_{11}H_{14}INO_2$: 319.01; Found $[M-H]^+$: 318.00.

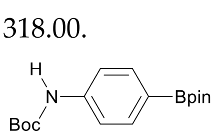

tert-Butyl (4-(4,4,5,5-tetramethyl-1,3,2-dioxaborolan-2-yl)-phenyl) carbamate (S9)

S8 (1.5 g, 4.70 mmol, 1 equiv), Bis(pinacolato)diboron (1.43 g, 5.64 mmol, 1.2 equiv), KOAc (2.31 g, 23.5 mmol, 5 equiv) and $PdCl_2(dppf)\cdot CH_2Cl_2$ (115 mg, 0.14 mmol, 3 mol%) were placed in a two-necked round bottom flask. The flask was then purged with nitrogen gas three times before addition of DMSO (45 mL). The mixture was stirred at 90 °C for 20 h.

After the reaction was complete, the mixture was extracted with EtOAc, washed with H_2O and brine successively. The organic layer was collected and dried over Na_2SO_4 and evaporated to dryness under reduced pressure. The crude sample was purified by silica gel column chromatography (EtOAc: hexane 1: 9) to yield target compound as white solid (1.43 g, 95%).

^{1}H-NMR (400 MHz, $CDCl_3$): δ = 7.73 (d, *J* = 8.6 Hz, 2H), δ = 7.36 (d, *J* = 8.8 Hz, 2H), δ = 6.52 (bs, 1H), δ = 1.52 (s, 9H), δ = 1.33 (s, 12H). ESI-FTMS (*m*/*z*) calcd for $C_{17}H_{26}BNO_4$: 319.20; Found $[M+Na]^+$: 342.19.

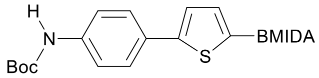

4-(Boc-amino) phenylthiophene-2-boronic MIDA ester (S10)

S7 (1.0 g, 3.15 mmol, 1 equiv), **S9** (1.21 g, 3.80 mmol, 1.2 equiv), K_3PO_4 (1.0 g, 9.42 mmol, 3 equiv), $PdCl_2$(dppf)· CH_2Cl_2 (103 mg, 0.13 mmol, 4 mol%) were placed in a two-necked round bottom flask. The flask was then purged with nitrogen gas three times before addition of THF (5 mL) and H_2O (0.28 mL, 15.75 mmol, 5 equiv). The mixture was stirred at r.t. for 24 h. The reaction mixture was diluted with acetone (5mL), dried over Na_2SO_4, filtrated through a pad Celite, washed with acetone, and then concentrated. The crude sample was purified by silica gel column chromatography (Acetone: Hexane 1:2) to yield the target compound as yellow solid (0.94 g, 75%).

^{1}H-NMR (400 MHz, ACETON-6*d*): δ = 8.52 (bs, 1H), δ = 7.61 (s, 4H), δ = 7.42 (d, *J* = 3.8 Hz, 1H), δ = 7.26 (d, *J* = 3.6 Hz, 1H), δ = 4.29 (d, *J* = 17 Hz, 2H), δ = 4.19 (d, *J* = 17 Hz, 2H), δ = 3.21 (s, 3H), δ = 1.50 (s, 9H). ESI-FTMS (*m*/*z*) calcd for $C_{20}H_{23}BN_2O_6S$: 430.14; Found $[M-H]^+$: 429.13.

© 2018 by the authors. Submitted for possible open access publication under the terms and conditions of the Creative Commons Attribution (CC BY) license (http://creativecommons.org/licenses/by/4.0/).
